# Draft Genome Sequence of Tubulinosema ratisbonensis, a Microsporidian Species Infecting the Model Organism Drosophila melanogaster

**DOI:** 10.1128/MRA.00077-19

**Published:** 2019-08-01

**Authors:** Valérie Polonais, Sebastian Niehus, Ivan Wawrzyniak, Adrien Franchet, Christine Gaspin, Abdel Belkorchia, Matthieu Reichstadt, Caroline Belser, Karine Labadie, Arnaud Couloux, Frédéric Delbac, Eric Peyretaillade, Dominique Ferrandon

**Affiliations:** a Université Clermont Auvergne, CNRS, Laboratoire Microorganismes: Génome et Environnement, Clermont-Ferrand, France; b Université de Strasbourg CNRS, M3I UPR 9022, Strasbourg, France; c Université de Toulouse, INRA, UR 875 Unité Mathématiques et Informatique Appliquées de Toulouse, Auzeville, Castanet-Tolosan, France; d INRA, Saint-Genès-Champanelle, France; e UMR1213 Herbivores, Clermont Université, VetAgro Sup, Clermont-Ferrand, France; f Genoscope, Institut de Biologie François-Jacob, Commissariat à l’Energie Atomique (CEA), Université Paris-Saclay, Evry, France; University of Maryland School of Medicine

## Abstract

We present the draft genome sequence of Tubulinosema ratisbonensis, a microsporidium species infecting Drosophila melanogaster. A total of 3,013 protein-encoding genes and an array of transposable elements were identified. This work represents a necessary step to develop a novel model of host-parasite relationships using the highly tractable genetic model D. melanogaster.

## ANNOUNCEMENT

Tubulinosema ratisbonensis belongs to the *Microsporidia*, a group of obligate intracellular parasites that group near the base of the fungal radiation (1). *T. ratisbonensis* is a pathogen of Drosophila melanogaster, responsible for fly culture collapse (2). This species presents a unique opportunity to study and better understand specific host-parasite interactions and also to decipher the adaptation capacities of this parasite. For example, a recent study demonstrated that phosphatidic acid is a limiting host metabolite for the proliferation of *T. ratisbonensis* in *Drosophila* flies (3).

The *T. ratisbonensis* strain isolated from D. melanogaster was propagated in the MRC-5 human cell line (4, 5). Spores were isolated and purified from host cells as previously described (6). *T. ratisbonensis* genomic DNA was extracted according to Katinka et al. (7), and two mate-paired libraries of 5-kb nebulized fragments were constructed to perform pyrosequencing as described by Aury et al. (8). 454 sequencing was used to remove low-quality sequences and generated 1,031,738 useful reads with an average size of 250 bp. In addition, genomic DNA was also randomly fragmented by sonication to produce an Illumina library according to Choulet et al. (9). An in-house quality control process based on the FastX package (http://www.genoscope.cns.fr/externe/fastxtend/) was applied to the reads that passed the Illumina quality filters defined by Alberti et al. (10). The 44,119,481 obtained single reads with an average length of 100 bp were assembled with pyrosequencing data using Newbler version 2.7 (11) with default parameters. To improve genome assembly, total RNAs were extracted from *Drosophila* S2R+ cells after 12 hours and 6 days postinfection with *T. ratisbonensis* using an RNeasy kit (Qiagen) according to the manufacturer’s protocol. Transcriptome sequencing (RNA-seq) Illumina libraries were then obtained as described by Lee et al. (12), and the 54,845,250 single-read sequences with an average length of 54 bp were assembled using the IDBA-UD assembler version 1.1.1 (13). Finally, *T. ratisbonensis* contigs obtained from whole-genome sequencing and RNA-seq approaches were assembled using the CAP3 program from the Geneious platform (14).

The resulting draft genome’s total size is 7,555,974 nucleotides (nt) spread over 952 contigs with an overall GC content of 23.2%. The *N*_50_ contig length is 12,152 bp, and the largest contig is composed of 59,712 bp. From these assembly data, a total of 3,013 coding DNA sequences (CDSs) were predicted using a dedicated microsporidian annotation pipeline (E. Peyretaillade, M. Reichstadt, S. Rimour, I. Wawrzyniak, and F. Delbac, unpublished data) combining homology search, transcriptional regulatory motif detection, and GLIMMER gene prediction (15, 16). Functional annotation conducted using Blast2GO software (17) with default parameters revealed that 2,099 CDSs (70%) present at least one ortholog in microsporidia, and 44.5% can be assigned to putative functions consistent with other microsporidium genomic data (18). Noncoding rRNA and tRNA genes were detected using RNammer (19) and tRNAScan-SE version 1.23 (20), respectively. Thus, six 5S rRNAs, a contig including the 16S-23S rRNA unit, and 55 tRNA genes were annotated. Additional noncoding RNAs were identified using a comparative analysis following a previously described approach (21) and Anncaliia algerae genomic data. Transposable elements (TEs) were obtained from tblastx analysis (E value cutoff of 1e-5) using TE sequences previously identified in numerous microsporidian species (22). An exhaustive search of TEs has also been carried out with TransposonPSI software (http://transposonpsi.sourceforge.net/) to identify highly variable subclasses of TEs. TE family affiliation was also checked using RepeatMasker (http://www.repeatmasker.org/). A total of 160 sequences were identified as TEs and were classified among the following 3 major families: long terminal repeat (LTR) retrotransposon (50.6%), DNA Mariner (20.6%), and DNA Merlin (20.6%). Among the 95 complete transposase sequences, 67.4% present a CCC/GGG-like signal upstream of the transcriptional initiation signals (16), suggesting that these sequences were under the control of *T. ratisbonensis* transcriptional machinery.

### Data availability.

Annotated contig sequences have been deposited at DDBJ/ENA/GenBank under the accession number RCSS00000000. The version described in this paper is version RCSS01000000. All the data produced for this project are available in NCBI databases under the accession numbers PRJEB31246 and PRJNA494796.
